# RNA-Seq Dataset From Isolated Leukocytes Following Spontaneous Intracerebral Hemorrhage in Zebrafish Larvae

**DOI:** 10.3389/fncel.2021.660732

**Published:** 2021-04-14

**Authors:** Siobhan Crilly, James Cooper, Lauren Bradford, Ian E. Prise, Siddharth Krishnan, Paul R. Kasher

**Affiliations:** ^1^Geoffrey Jefferson Brain Research Centre, Manchester Academic Health Science Centre, Northern Care Alliance, University of Manchester, Manchester, United Kingdom; ^2^Division of Neuroscience and Experimental Psychology, Faculty of Biology, Medicine and Health, School of Biological Sciences, University of Manchester, Manchester, United Kingdom; ^3^Faculty of Biology, Medicine and Health, Manchester Academic Health Science Centre, Lydia Becker Institute of Immunology and Inflammation, University of Manchester, Manchester, United Kingdom; ^4^Division of Infection, Immunity and Respiratory Medicine, Faculty of Biology, Medicine and Health, School of Biological Sciences, University of Manchester, Manchester, United Kingdom

**Keywords:** RNA sequence analysis, zebrafish, leukocytes, intracerebral hemorrhage, translational research

## Introduction

The pathogenesis of intracerebral hemorrhage (ICH) can be divided into two distinct injury phases. Almost immediately following ICH, mechanical tissue disruption and mass effect induces primary brain injury, followed by an activated immune response to damaged brain cells and products of red blood cell lysis that drives a secondary wave of brain injury. Secondary brain injury causes increased intracranial pressure and worse pathological outcomes in both pre-clinical models and in ICH patients (Wang, 2010). Modulating the immune response represents a realistic target for therapeutics after ICH, however to date a precise understanding of the specific neuroinflammatory landscape in ICH is currently lacking. Although anti-inflammatory trials for ICH have started, these are based on observations from ischemic stroke and subarachnoid hemorrhage trials (Galea et al., 2017; BLOC-ICH, 2018; Smith et al., 2018). Using transcriptomic profiling to improve our fundamental understanding of the molecular regulation of inflammation after ICH could reveal new therapeutic strategies for patients to improve clinical outcomes after stroke.

Leukocytes can contribute to both neuroprotection and neurodegeneration after ICH-induced brain injury. Following primary injury, extravasation of leukocytes into the brain parenchyma contributes to a rise in intracranial pressure and oedema. Activated innate immune cells, such as macrophages and neutrophils, are recruited from the periphery to the brain and generate free radicals that contribute to oxidative damage of neurons (Zhao et al., 2007). Conversely, activated microglia and brain macrophages, are also essential for haematoma clearance and recovery after ICH (Zhao et al., 2015). Studying the transcriptomic profile in these cell types will contribute to our understanding of how these cells are regulated following ICH and may highlight specific molecular targets to modulate the inflammatory response to benefit patients.

**Figure 1:**
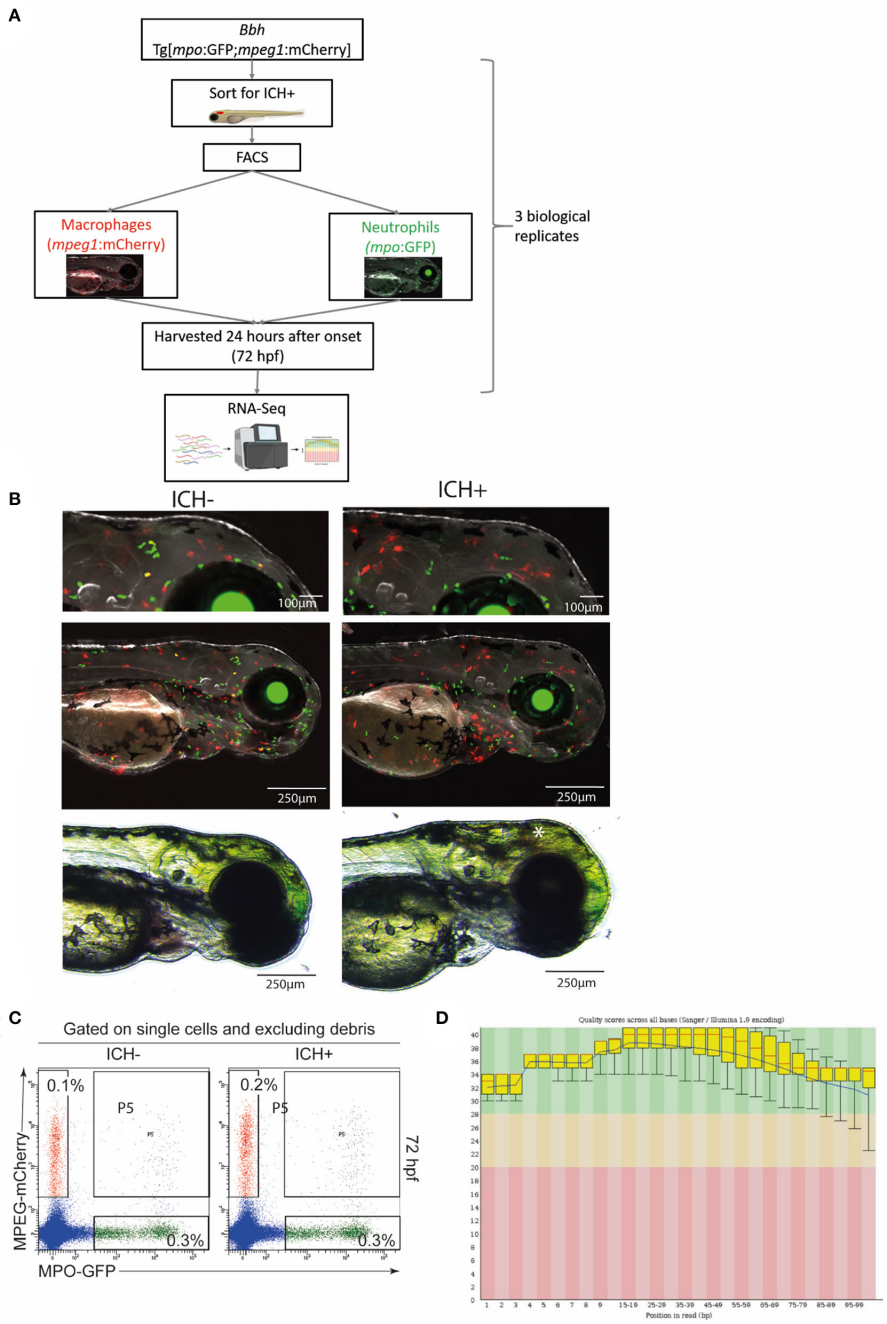
Experimental workflow and quality control steps for transcriptomic analysis. **(A)** Diagrammatical representation of the sample acquisition and preparation. *Bbh, Bubblehead* mutants; *mpo*, myeloperoxidase; GFP, green fluorescent protein; *mpeg1*, macrophage expressed gene 1; mCherry, red fluorescent protein; ICH+, hemorrhaged larvae; ICH–, non-hemorrhaged sibling controls; hpf, hours post fertilization; FACS, fluorescence-activated cell sorting; RNA-Seq, RNA sequencing. **(B)** Representative images of ICH– and ICH+ mpo:mpeg larvae at 72 hpf. Larvae were anesthetized briefly and images were acquired using the Leica M205 FA Stereo fluorescence microscope. Larvae were confirmed to have expression of green *mpo* positive neutrophils and red *mpeg1* positive macrophages, and separated according to presence of ICH (*). Images at higher magnification (top panels) shows an increase in *mpeg1* positive cells within the brain as previously observed. **(C)** Sort strategy to isolate macrophages and neutrophils from ICH+ and ICH– control zebrafish larvae at 72 hpf. From a single cell suspension, neutrophils were sorted based on their expression of GFP and macrophages by their expression of mCherry. Cells expressing both (P5) were excluded from all analyses. Numbers represent frequencies of macrophages and neutrophils as a proportion of viable cells. Plots are representative from three independent replicates. **(D)** FastQC plots for the quality across all bases from all 12 samples. Graphical representation of the quality for each base pair position in the reads. Green region equates to very good quality calls, orange is reasonable and red poor quality. Box plots show the median value and interquartile range, whiskers include 10 and 90% points. Blue line is overall mean quality.

**Figure 2:**
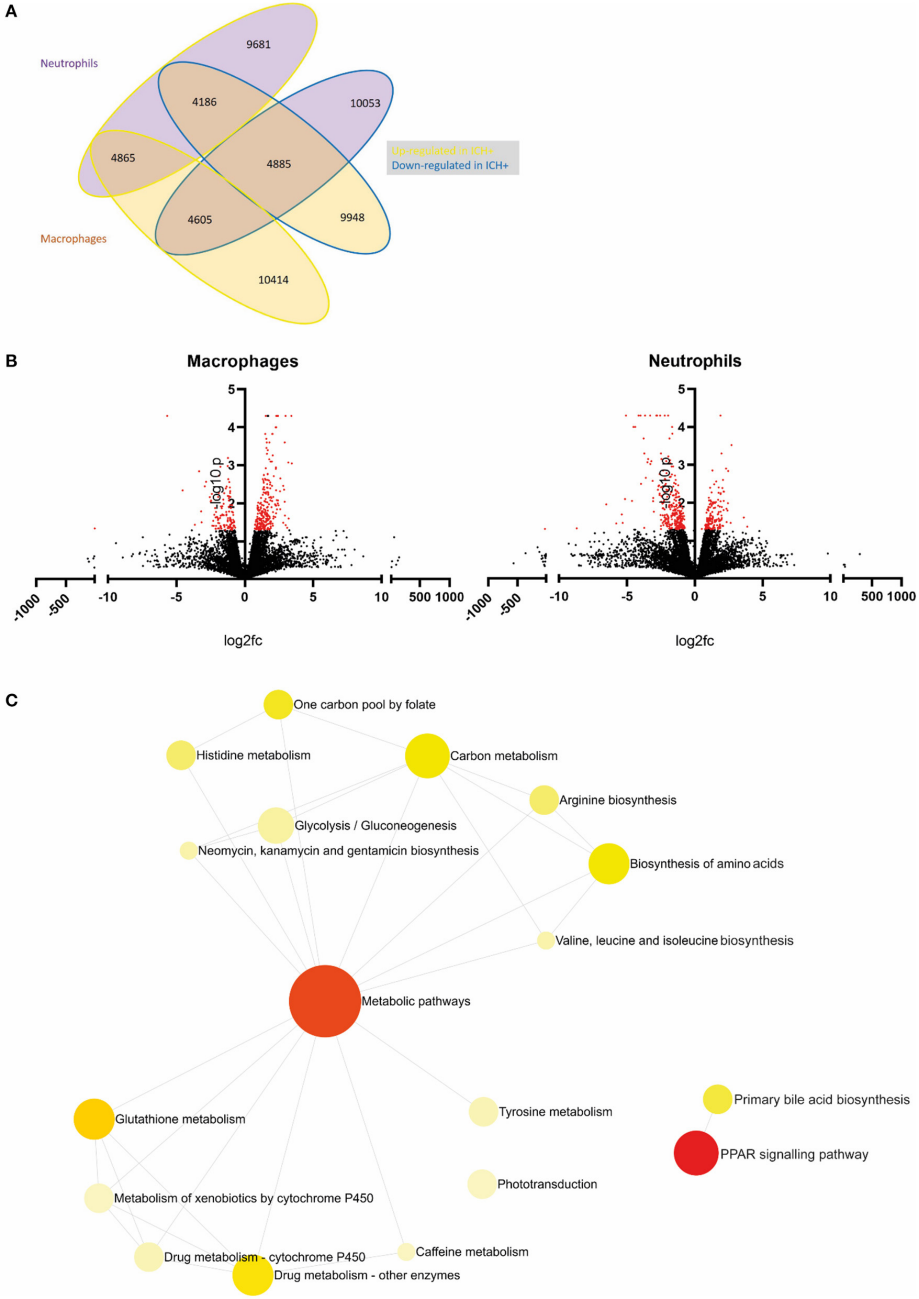
An example of primary analysis suggests metabolic pathways are dysregulated in leukocytes following ICH in zebrafish larvae. **(A)** Summary of gene expression across leukocyte samples in response to hemorrhage. Values outlined in yellow are upregulated and those outlined in blue are downregulated in hemorrhage. Values of genes expressed in neutrophils are in purple bubbles and those in macrophages are in orange bubbles. **(B)** Volcano plots showing the log2 fold change of gene expression in macrophages (left panel) and neutrophils (right panel) when compared to ICH– samples. Significant genes (*p* < 0.05, log10 > 1.3) are in red. **(C)** Network analysis reveals dysregulation in metabolic pathways in leukocytes following ICH. Metabolic pathways and PPAR signaling pathway pAdj = 0.004.

Here, we employed a zebrafish larval model of spontaneous ICH (*bubblehead; bbh*) to identify a transcriptomic signature of the innate immune response in the secondary injury phase following ICH. The *bbh* mutant has a hypomorphic mutation in β*pix*, resulting in leaky neurovasculature at a critical developmental timepoint (Liu et al., 2007). Zebrafish larvae represent a useful alternative *in vivo* system for studying ICH pre-clinically. Unlike more commonly used rodent models, ICH in *bbh* mutant zebrafish larvae can occur without the need for invasive surgical procedures and thereby mimic the spontaneous nature of the human condition more closely. We have previously characterized disease outcomes in the zebrafish *bbh* model which recapitulates key pathological outcomes observed in mammalian models and patients (Crilly et al., 2018, 2019). The secondary injury response in zebrafish larvae is identified by an increase in macrophage recruitment and activation within the head at 24 h post injury, whilst neutrophils are the most numerous leukocyte population at this developmental stage. Using a double transgenic zebrafish reporter line crossed onto the mutant *bbh* background, we isolated macrophages and neutrophils from this period of secondary injury 24 h after ICH and performed transcriptomic profiling using RNA-Seq analyses (Figure 1). Data acquired in this study showed differential gene expression in leukocytes between ICH– and ICH+ groups, in addition to the differences between the two cell types (Figure 2).

Transcriptomic approaches have been performed by others using clinical ICH samples (Dykstra-Aiello et al., 2015; Sang et al., 2017) which are mostly acquired from peripheral blood, but most recently include longitudinal transcriptomic profiling in myeloid cells obtained from haematoma samples (Askenase et al., 2021). We propose that our zebrafish dataset can be used to make cell-specific, cross-species comparisons to strengthen our understanding of the evolutionarily conserved transcriptional changes that occur in innate immune cells following spontaneous ICH and to highlight translationally relevant candidate molecular targets for the future treatment of the disease.

### Methods

#### Zebrafish (Danio Rerio) Strains

Zebrafish were raised and maintained at The University of Manchester Biological Services Unit under standard conditions as previously described (Westerfield, 2000). Adult zebrafish husbandry was approved by the University of Manchester Animal Welfare and Ethical Review Board. All experiments were performed in accordance with U.K. Home Office regulations (PPL:P132EB6D7) and reported according to ARRIVE guidelines (Percie du Sert et al., 2020). Transgenic lines used in this study include the macrophage-specific lineage *mpeg1:*mCherry [constructed in-house as previously described (Ellett et al., 2011)] and the neutrophil-specific *mpo:*GFP (Renshaw et al., 2006), crossed onto the mutant *bbh*^m292^ (Liu et al., 2007) background. Fertilized embryos were collected from natural spawning and incubated at 28.5°C in standard E3 embryo medium and staged according to standard guidelines (Kimmel et al., 1995). Zebrafish larvae were terminated at 72 h post fertilization (hpf) using a lethal overdose of 4% MS222 anesthesia prior to tissue dissociation. Surplus embryos were terminated and confirmed dead by freezing at −20°C.

#### ICH Model

Larvae from heterozygous adult *bbh* Tg[*mpo:*GFP;*mpeg1:*mcherry] (referred to as mpo:mpeg) in-crosses were collected and light microscopy used to identify brain hemorrhages (ICH+) in homozygotes, which were separated from non-hemorrhaged (ICH–) heterozygous and wild-type siblings at ~52 hpf. At 72 hpf, 24 h after injury, larvae were collected for tissue dissociation.

#### Image Acquisition

To verify the presence of double transgenic expression and brain hemorrhage, larvae were screened at 72 hpf using a Leica M205 FA Stereo fluorescence microscope and a DFC365FX camera. *Bbh* mpo:mpeg ICH– and ICH+ groups were anesthetised briefly using 0.2% MS222 and expression of both GFP-positive neutrophils and mCherry-positive macrophages was confirmed. Representative images were acquired (Figure 1B) at 100X and 35X visual magnification with 1X optical objective and processed with LAS X software (V1.0.10).

#### Tissue Preparation

Larvae were collected as three biological replicates at 72 hpf. To isolate specific immune cell populations, anesthetized groups of ICH- and ICH+ mpo:mpeg double transgenic embryos (*n* = 250 each group, per replicate) were washed twice in PBS and lysed using 250 μl 10X TrypLE (Life Technologies) and 250 μl 10 mg/ml collagenase/dispase (Roche) per 100 embryos. Samples were incubated at 28.5°C for 30 min and triturated repeatedly. 5 μl DNaseI (Invitrogen) and 50 μl of 10X DNaseI buffer was added and the solution was incubated for a further 30 min until a single cell suspension was obtained. Cells were isolated and resuspended in 500 μl PBS with DNaseI before sorting for fluorescence.

#### Fluorescence-Activated Cell Sorting

Single cell suspensions were run on the BD FACSAria Fusion at The University of Manchester Flow Cytometry Core facility. Approximately 1,000–3,000 cells expressing mCherry (macrophages) and GFP (neutrophils) were separated after excluding debris, dead cells by FSC-SSC and doublets. Cells were sorted directly into RLT (Qiagen) with β-mercaptoethanol (Life Technologies) at a 10:1 ratio for downstream processing.

#### RNA Extraction and Sequencing Analysis

In order to extract RNA from samples with low cell numbers, a single cell RNA extraction kit (Norgen Biotek Corp P4-0164) was used to elute 8 μl of purified RNA according to manufacturer's instructions. Samples (*n* = 3) from both ICH– and ICH+ conditions for isolated macrophages and neutrophils were shipped on dry ice to Theragen Etex Bio Institute in South Korea for sequencing. Briefly, quality control was performed and cDNA libraries were generated using the SMART-Seq V4 Ultra Low Input RNA Kit (Takara Bio) and samples were sequenced on the NovaSeq 6000 15-bp Pair End platform. Analysis for comparative expression was performed in-house using Ingenuity Pathway Analysis (Qiagen). Reads were filtered with Trimmomatic (v0.36) (Bolger et al., 2014). Filtered fastq files were aligned to the zebrafish GENCODE genome (GRCz11) using STAR (v2.5.3) (Dobin et al., 2013). Filtered reads were then sorted, compressed, and unaligned reads removed using samtools (v1.3) (Li et al., 2009; Li, 2011). Aligned reads were then counted, normalized, and compared, respectively, using the Cuffquant, Cuffnorm, and Cuffdiff function of Cufflinks (v2.2.2) (Trapnell et al., 2010).

#### Data Analysis

Statistical analysis was carried out using Ingenuity Pathway Analysis software (Qiagen) to determine *p*-value significance and q value false discovery rate (Krämer et al., 2014). Data presentation was performed using GraphPad Prism (v8.1.2) in Figure 2B. Gene ontology enrichment analysis was carried out and visualized using Networks Analyst 3.0 (https://www.networkanalyst.ca/), to determine enriched biological pathways. Genes that were differentially expressed (*p* < 0.05 and q <0.05) in leukocytes (neutrophils−139 genes; macrophages−63 genes) from ICH+ larvae when compared to ICH– are visualized in Figure 2C. The full list of genes is presented in shared file doi: 10.6084/m9.figshare.13664606(CrillyCooper2021.xlsx).

### Data Records

The full RNA-Seq dataset is available on the figshare data sharing platform doi: 10.6084/m9.figshare.13664606. Data from the Theragen Etex Bioinformatics team are presented in an.xlsx file and contains raw expression data readings from each repeat in both leukocyte samples. Sheets containing gene name information corresponding to the ensembl gene ID for all 32,053 identified genes is included. Further sheets provide lists of significant differentially expressed genes in neutrophils and macrophages, respectively.

### Technical Validation

#### Phenotypic Analysis of Larvae

To confirm the presence of hemorrhage at 52 hpf and the expression of transgenic genes, larvae were visualized using fluorescence stereomicroscopy for screening. Homozygous *bbh* mutant larvae hemorrhage spontaneously at ~33 hpf, and blood is visible using non-invasive light microscopy until ~100 hpf. Representative images of mpo:mpeg double positive ICH+ larvae are shown (Figure 1B) Only larvae with confirmed ICH and double transgenic expression were selected for tissue digestion and analysis. Surplus larvae were terminated as outlined above.

#### Quality Control of FACS Analysis

To ensure the separation of transgenic cells into populations of different leukocytes, single, live cells were selected based on expression of mCherry (macrophages) or GFP (neutrophils). Data was gated to exclude debris and doublets and quality analysis (Figure 1C) shows exclusion of double transgenic singlets.

#### Quality Assessment of RNA and RNA-Seq Data

A low overall RNA yield from isolated cells resulted in use of the SMART-Seq Ultra Low Input RNA kit (Takara Bio) for cDNA synthesis, amplification, purification, and validation. This protocol was optimized for between 10 pg and 10 ng of RNA. All samples were checked for quality control prior to quantitative analysis. Sample information (Supplementary Table 1) shows total number of reads and bases expressed for each sample, including a Q30 MoreBasesRate showing the proportion of nucleotides greater than Phred quality score of 30. Additional quality checks were carried out by FastQC Software and overall quality scores across all bases is shown (Figure 1D).

### Primary Analysis

Primary analysis of our data revealed a list of genes which are dysregulated in leukocytes after ICH when compared to ICH– controls. Visualization of these data in Figure 2A shows macrophages appear to upregulate expression of more genes in response to hemorrhage (10414) compared to neutrophils (9681) and less than half of these genes are shared between cell types (4865). Figure 2B shows all dysregulated genes in a volcano plot separated for each cell type. Significantly differentially expressed genes (*p* < 0.05, log10 > 1.3) are shown in red. Network analysis of the pathways implicated by these significantly differentially expressed genes in Figure 2C suggests that ICH injury leads to a considerable dysregulation of metabolic processes in leukocytes. Metabolism of glucose is closely linked to function in myeloid cells (Jung et al., 2019), regulation of bile acids has previously been linked clinically (Wang et al., 2018), and pre-clinically (Rodrigues et al., 2002, 2003) to neuroprotection in ICH and amino acid biosynthesis may imply a switch to a pro-regenerative transcriptional profile. Recent work in transcriptomic profiling of patient leukocytes shows cells undergo significant metabolic reprogramming over the acute and subacute ICH injury timeline (Askenase et al., 2021). These raw data and preliminary analyses suggest a number of genes and subsequent pathways for further investigation in this model to elucidate function following ICH, and will potentially provide a scientific basis for mechanistic investigation in alternative pre-clinical models, and support translational comparisons to patient data.

### Usage Notes

We suggest that further analysis of these data for gene ontology terms is performed using Ingenuity Pathway Analysis (Qiagen) software, or similar free software available online to identify genes and/or molecular pathways that could be studied further in the context of translational ICH research. Data can be visualized as a heat map using Morpheus (Broad Institute). Variability between biological replicates is observed in zebrafish datasets due to underlying genetic heterogeneity of a relatively outbred population. Similarly to the ImmGen Consortium (Harvard) and the Human Cell Atlas project, these data can aid cross-species comparisons in health and disease.

There can also be distinct differences between clutch samples (batches of zebrafish eggs), and this should be considered as a random variable for statistical analysis. For these reasons, identifying significant genes using *p*-adjusted value alone is not recommended, there are many factors that contribute to the significance of this value. Considering the quality and consistency of the reads between each sample, use of the *p*-value and the fold change are all recommended to assess changes in expression levels.

We propose this dataset will provide a valuable resource for the translational ICH research community and allow for cross-species comparisons. This could include, for example, alignment with RNAseq datasets generated from samples obtained from rodent models or ICH patients. Such comparisons will allow us to identify evolutionarily conserved inflammatory regulators of injury following spontaneous ICH, and highlight candidate therapeutic target genes and/or pathways. When comparing datasets between model species it is important to note the speed of development in the zebrafish model, and that 24 h after injury in this model might be equivalent to later time points in both rodent models and patients. The peak of the immune response observed pre-clinically varies with type of ICH model, species and co-morbidities (Mracsko and Veltkamp, 2014). In patients and clinical samples the inflammatory response is sustained to 21 days after injury (Aronowski and Zhao, 2011; Tschoe et al., 2020). Furthermore, transcriptional differences may exist based on the cellular source of RNA used for sequencing. Therefore, any future cross-species transcriptional comparisons need to take relative brain injury phase differences and cell/tissue type into account.

## Data Availability Statement

The datasets presented in this study can be found in online repositories. The names of the repository/repositories and accession number(s) can be found in the article/Supplementary Material.

## Ethics Statement

The animal study was reviewed and approved by University of Manchester Animal Welfare and Ethical Review Board.

## Author Contributions

JC and SC acquired and prepared the samples and carried out the experimental work. IEP and LB carried out data analysis. SC, SK, and PRK wrote and reviewed the manuscript. PRK is responsible for acquiring the funding for the study. All authors contributed to the article and approved the submitted version.

## Conflict of Interest

The authors declare that the research was conducted in the absence of any commercial or financial relationships that could be construed as a potential conflict of interest.

## Supplementary Material

Supplementary Table 1 Sample read and quality information.

## References

[B1] Aronowski J. Zhao X. (2011). Molecular pathophysiology of cerebral hemorrhage: secondary brain injury. Stroke 42, 1781–1786. 10.1161/STROKEAHA.110.596718 21527759 PMC3123894

[B2] Askenase M. H. Goods B. A. Beatty H. E. Steinschneider A. F. Velazquez S. E. Osherov A.. (2021). Longitudinal transcriptomics define the stages of myeloid activation in the living human brain after intracerebral hemorrhage. Sci. Immunol. 6:eabd6279. 10.1126/sciimmunol.abd6279 33891558 PMC8252865

[B3] BLOC-ICH (2018). Interleukin-1 Receptor Antagonist in Intracerebral Haemorrhage (BLOC-ICH). Available online at: https://clinicaltrials.gov/ct2/show/NCT03737344 (accessed January 29, 2021).

[B4] Bolger A. M. Lohse M. Usadel B. (2014). Trimmomatic: a flexible trimmer for Illumina sequence data. Bioinformatics 30, 2114–2120. 10.1093/bioinformatics/btu170 24695404 PMC4103590

[B5] Crilly S. Njegic A. Laurie S. E. Fotiou E. Hudson G. Barrington J.. (2018). Using zebrafish larval models to study brain injury, locomotor, and neuroinflammatory outcomes following intracerebral haemorrhage. F1000Research 7:1617. 10.12688/f1000research.16473.2 30473780 PMC6234746

[B6] Crilly S. Njegic A. Parry-Jones A. R. Allan S. M. Kasher P. R. (2019). Using zebrafish larvae to study the pathological consequences of hemorrhagic stroke. J. Vis. Exp. e59716. 10.3791/59716. [Epub ahead of print]. 31233021

[B7] Dobin A. Davis C. A. Schlesinger F. Drenkow J. Zaleski C. Jha S.. (2013). STAR: ultrafast universal RNA-seq aligner. Bioinformatics 29, 15–21. 10.1093/bioinformatics/bts635 23104886 PMC3530905

[B8] Dykstra-Aiello C. Jickling G. C. Ander B. P. Zhan X. Liu D. Hull H.. (2015). Intracerebral hemorrhage and ischemic stroke of different etiologies have distinct alternatively spliced mRNA profiles in the blood: a pilot RNA-seq study. Transl. Stroke Res. 6, 284–289. 10.1007/s12975-015-0407-9 25994285 PMC4485700

[B9] Ellett F. Pase L. Hayman J. W. Andrianopoulos A. Lieschke G. J. (2011). mpeg1 promoter transgenes direct macrophage-lineage expression in zebrafish. Blood 117, e49–e56. 10.1182/blood-2010-10-314120 21084707 PMC3056479

[B10] Galea J. Ogungbenro K. Hulme S. Patel H. Scarth S. Hoadley M.. (2017). Reduction of inflammation after administration of interleukin-1 receptor antagonist following aneurysmal subarachnoid hemorrhage: results of the subcutaneous interleukin-1Ra in SAH (SCIL-SAH) study. J. Neurosurg. 28, 515–523. 10.3171/2016.9.JNS16615 28298024

[B11] Jung J. Zeng H. Horng T. (2019). Metabolism as a guiding force for immunity. Nat. Cell Biol. 21:85. 10.1038/s41556-018-0217-x 30602764

[B12] Kimmel C. B. Ballard W. W. Kimmel S. R. Ullmann B. Schilling T. F. (1995). Stages of embryonic development of the zebrafish. Dev. Dyn. 203, 253–310. 10.1002/aja.1002030302 8589427

[B13] Krämer A. Green J. Pollard Jr J. Tugendreich S. (2014). Causal analysis approaches in ingenuity pathway analysis. Bioinformatics 30, 523–530. 10.1093/bioinformatics/btt703 24336805 PMC3928520

[B14] Li H. (2011). A statistical framework for SNP calling, mutation discovery, association mapping, and population genetical parameter estimation from sequencing data. Bioinformatics 27, 2987–2993. 10.1093/bioinformatics/btr509 21903627 PMC3198575

[B15] Li H. Handsaker B. Wysoker A. Fennell T. Ruan J. Homer N.. (2009). The sequence alignment/map format and SAMtools. Bioinformatics 25, 2078–2079. 10.1093/bioinformatics/btp352 19505943 PMC2723002

[B16] Liu J. Fraser S. D. Faloon P. W. Rollins E. L. Vom Berg J. Starovic-Subota O.. (2007). A βPix–Pak2a signaling pathway regulates cerebral vascular stability in zebrafish. Proc. Natl. Acad. Sci. U.S.A. 104, 13990–13995. 10.1073/pnas.0700825104 17573532 PMC1955796

[B17] Mracsko E. Veltkamp R. (2014). Neuroinflammation after intracerebral hemorrhage. Front. Cell. Neurosci. 8:388. 10.3389/fncel.2014.00388 25477782 PMC4238323

[B18] Percie du Sert N. Hurst V. Ahluwalia A. Alam S. Avey M. T. Baker M.. (2020). The ARRIVE guidelines 2.0: updated guidelines for reporting animal research. PLoS Biol. 18:e3000410. 10.1371/journal.pbio.3000410 32663219 PMC7360023

[B19] Renshaw S. A. Loynes C. A. Trushell D. M. Elworthy S. Ingham P. W. Whyte M. K. (2006). A transgenic zebrafish model of neutrophilic inflammation. Blood 108, 3976–3978. 10.1182/blood-2006-05-024075 16926288

[B20] Rodrigues C. M. Solá S Nan Z. Castro R. E. Ribeiro P. S. Low W. C.. (2003). Tauroursodeoxycholic acid reduces apoptosis and protects against neurological injury after acute hemorrhagic stroke in rats. Proc. Natl. Acad. Sci. U.S.A. 100, 6087–6092. 10.1073/pnas.1031632100 12721362 PMC156330

[B21] Rodrigues C. M. Spellman S. R. Solá S Grande A. W. Linehan-Stieers C. Low W. C.. (2002). Neuroprotection by a bile acid in an acute stroke model in the rat. J. Cereb. Blood Flow Metab. 22, 463–471. 10.1097/00004647-200204000-00010 11919517

[B22] Sang M. Wang X. Zhang H. Sun X. Ding X. Wang P.. (2017). Gene expression profile of peripheral blood mononuclear cells in response to intracerebral hemorrhage. DNA Cell Biol. 36, 647–654. 10.1089/dna.2017.3650 28654306

[B23] Smith C. J. Hulme S. Vail A. Heal C. Parry-Jones A. R. Scarth S.. (2018). SCIL-STROKE (Subcutaneous Interleukin-1 Receptor Antagonist in Ischemic Stroke): a randomized controlled phase 2 trial. Stroke 49, 1210–1216. 10.1161/STROKEAHA.118.020750 29567761

[B24] Trapnell C. Williams B. A. Pertea G. Mortazavi A. Kwan G. Van Baren M. J.. (2010). Transcript assembly and quantification by RNA-Seq reveals unannotated transcripts and isoform switching during cell differentiation. Nat. Biotechnol. 28, 511–515. 10.1038/nbt.1621 20436464 PMC3146043

[B25] Tschoe C. Bushnell C. D. Duncan P. W. Alexander-Miller M. A. Wolfe S. Q. (2020). Neuroinflammation after intracerebral hemorrhage and potential therapeutic targets. J. Stroke 22:29. 10.5853/jos.2019.02236 32027790 PMC7005353

[B26] Wang J. (2010). Preclinical and clinical research on inflammation after intracerebral hemorrhage. Progr. Neurobiol. 92, 463–477. 10.1016/j.pneurobio.2010.08.001 20713126 PMC2991407

[B27] Wang K. Zhang Y. Zhong C. Zheng D. Xu J. Zhang Y.. (2018). Increased serum total bile acids can be associated with a small hematoma volume and decreased clinical severity during acute intracerebral hemorrhage. Curr. Neurovasc. Res. 15, 158–163. 10.2174/1567202615666180516114211 29766806

[B28] Westerfield M. (2000). The Zebrafish Book: A Guide for the Laboratory Use of Zebrafish. Available online at: http://zfinorg/zf_info/zfbook/zfbkhtml (accessed January 29, 2021).

[B29] Zhao X. Sun G. Ting S. M. Song S. Zhang J. Edwards N. J.. (2015). Cleaning up after ICH: the role of Nrf2 in modulating microglia function and hematoma clearance. J. Neurochem. 133, 144–152. 10.1111/jnc.12974 25328080 PMC4361377

[B30] Zhao X. Sun G. Zhang J. Strong R. Song W. Gonzales N.. (2007). Hematoma resolution as a target for intracerebral hemorrhage treatment: role for peroxisome proliferator-activated receptor γ in microglia/macrophages. Ann. Neurol. 61, 352–362. 10.1002/ana.21097 17457822

