# Supplementary material for: RNA-Seq Dataset From Isolated Leukocytes Following Spontaneous Intracerebral Hemorrhage in Zebrafish Larvae
Source: Front Cell Neurosci. 2021 Apr 14;15:660732. doi: 10.3389/fncel.2021.660732 (PMC8079741; doi:10.3389/fncel.2021.660732)
Supplement: Supplementary Table 1 — Sample read and quality information. [file Table_1.DOCX]

| **Sample** | **Total reads** | **Total bases (bp)** | **Total bases (Gbp)** | **GC percent** | **Q30 MoreBasesRate** |
| --- | --- | --- | --- | --- | --- |
| H+ Neutrophils R1 | 79,523,894 | 12,008,107,994 | 12.01 | 48.20% | 92.88% |
| H+ Macrophages R1 | 80,654,354 | 12,178,807,454 | 12.18 | 48.08% | 92.80% |
| H- Neutrophils R1 | 81,305,382 | 12,277,112,682 | 12.28 | 48.00% | 92.52% |
| H- Macrophages R1 | 81,751,324 | 12,344,449,924 | 12.34 | 48.15% | 92.47% |
| H+ Neutrophils R2 | 80,852,206 | 12,208,683,106 | 12.21 | 48.68% | 93.15% |
| H+ Macrophages R2 | 81,374,968 | 12,287,620,168 | 12.29 | 48.96% | 92.81% |
| H- Neutrophils R2 | 81,633,426 | 12,326,647,326 | 12.33 | 48.69% | 93.03% |
| H- Macrophages R2 | 81,936,264 | 12,372,375,864 | 12.37 | 48.43% | 92.40% |
| H+ Neutrophils R3 | 80,981,804 | 12,228,252,404 | 12.23 | 49.11% | 93.08% |
| H+ Macrophages R3 | 79,622,662 | 12,023,021,962 | 12.02 | 47.61% | 92.75% |
| H- Neutrophils  R3 | 80,248,102 | 12,117,463,402 | 12.12 | 49.25% | 92.70% |
| H- Macrophages  R3 | 80,699,500 | 12,185,624,500 | 12.19 | 47.33% | 93.01% |
